# A Proposal for a New Classification of the Supernumerary Heads of the Biceps Brachii Muscle

**DOI:** 10.1155/2022/1510363

**Published:** 2022-04-22

**Authors:** Bartłomiej Szewczyk, Jose Ramon Sanudo, Michał Podgórski, Nicol Zielinska, Maria Bettencourt Pires, Paloma Aragonés, Łukasz Olewnik

**Affiliations:** ^1^Department of Anatomical Dissection and Donation, Medical University of Lodz, Lodz, Poland; ^2^Department of Human Anatomy and Embryology, Facultad de Medicina, Universidad Complutense de Madrid, Spain; ^3^Department of Diagnostic Imaging, Polish Mother's Memorial Hospital-Research Institute, Łódź, Poland; ^4^Department of Surgery and Human Morphology, Universidade NOVA de Lisboa, Lisbon, Portugal; ^5^Department of Orthopedics Surgery, Hospital Santa Cristina, Madrid, Spain

## Abstract

**Introduction:**

The anterior compartment of the arm consists of three muscles: the biceps brachii (BB), brachialis, and coracobrachialis muscle. The aim of the present study was to characterize possible variations in the supernumerary heads of the biceps brachii and use these to prepare an accurate classification of the area that could be used for planning surgical procedures in the region. *Material and Methods*. One hundred (51 left and 49 right, 52 females and 48 males) upper limbs fixed in 10% formalin solution were examined.

**Results:**

Four types of supernumerary BB heads were identified, with subtypes. Type I was the most common type, characterized by the two heads (64%); this was subdivided into Type IA, with a single muscle belly, and Type IB with two muscle bellies. The second most common type was Type II, which was characterized by the three BB heads (26%). This type was divided into four subtypes (A-D): Type IIa characterized by attachment to the middle part of the shaft of the humerus; Type IIb characterized by the origin to the coracoid process together with the short head of the BB; Type IIc characterized by origin to the tendon of the pectoralis major muscle; and Type IId characterized by the attachment to the capsule of the humeral joint. The third most common type was Type III, which was characterized by four heads (6%); this was divided into Type IIIa, where two heads originated from the humerus bone, and Type IIIb, where one head originated from the short heads and the second from the long head of the BB. The rarest type was Type IV (4%) which was characterized by five heads: the short head originated from the coracoid process and the long head originated from the supraglenoid tubercle, the third and fourth head originated from the shaft of the humerus, while the fifth head originated from the pectoralis major muscle.

**Conclusion:**

The biceps brachii is characterized by very high morphological variability. The new classification proposes four types of supernumerary head arrangement (I-IV), divided into subtypes. This classification has both clinical and anatomical significance.

## 1. Introduction

One of the muscles of the anterior compartment of the arm is the biceps brachii muscle (BB). The BB has two heads: a short head originating at the coracoid process and a long head, originating at the supraglenoid tubercle of the scapula. From its origin on the glenoid, the long head remains tendinous as it passes through the shoulder joint and through the intertubercular groove of the humerus. Extending from its origin on the coracoid, the tendon of the short head runs adjacent to the tendon of the coracobrachialis as the conjoint tendon. Both heads of the BB join in the middle of the upper arm to form a single muscle mass, usually near the insertion of the deltoid, to form a common muscle belly. In the distal part, the common muscle belly forms two components, the first being a tendon that inserts into the radial tuberosity and the second component being the lacertus fibrosus (LF) which fuses with the fascia of the forearm flexors [1].

The BB frequently demonstrates morphological variations which concern both the proximal attachment and distal attachment, as well as the frequency of additional heads [2–13]. However, the greatest degree of morphological variations arguably concerns the number of supernumerary heads, which can range from three to five [2, 7] In addition, accessory heads can arise from the deltoid muscle, pectoralis major, the coracoid process, or the humerus [3, 4, 7, 10, 14–17]. The supernumerary head of the BB has functional and clinical implications arising from their potential to influence certain muscle functions or induce symptoms of neurovascular compression [3].

Although many studies have examined the morphological variability of the accessory heads of the BB, there remains a need for an effective classification. Therefore, the aim of this work is to create the first accurate classification of the accessory heads of the BB.

## 2. Material and Methods

One hundred and one upper limbs (51 left and 49 right; 52 female and 48 male) fixed in 10% formalin solution were examined. The mean age of the cadavers at death was 77.9 years (range 53-95) (Central European population). The cadavers were obtained through deliberate donation to the Medical University anatomy program. Any upper limbs with evidence of surgical intervention in the dissected area were excluded. All dissection procedures in the shoulder and arm area were performed in accordance with a preestablished protocol [18–25].

Dissection began with the removal of the skin and superficial fascia from the area of the shoulder, thorax (pectoralis major area), the anterior and medial side of the arm, and the anterior side of the forearm. The next step was to dissect proximal insertion of pectoralis major muscle and remove it from area of thorax. This allowed the lateral, medial, and posterior cords of the brachial plexus to be visualized, as well as both biceps brachii, coracobrachialis, and brachialis muscles. Following this, the site of the lacertus LF was carefully checked. After checking and measuring the LF, the muscles of the anterior forearm group were identified to locate and measure the tendons and examine their insertion. Next, all the structures were thoroughly cleaned.

Upon dissection, the following morphological features of the BB were assessed:

- The morphology of the BB
- The accessory heads of the BB
- Some morphometric measurements of the BB

BB dissection was performed in accordance with the following principles:

- When clearing the BB, attention was paid to the presence of any accessory heads
- When cleaning the accessory heads, care was taken not to damage the musculocutaneous nerve, median nerve, or ulnar nerve, which may run between the accessory heads
- When cleaning the accessory heads, care was taken not to damage the brachial artery or branches of the brachial artery, which may run between the accessory heads
- When cleaning the accessory heads, care was taken not to tear the rare coracobrachialis longus muscle, which occurs in the distal part of the arm [18, 23]

An electronic digital caliper was used for all measurements (Mitutoyo Corporation, Kawasaki-shi, Kanagawa, Japan), and each measurement was performed twice with an accuracy of up to 0.1 mm. The study protocol was approved by the Bioethics Committee of the Medical University of Lodz (resolution RNN/1337/20/KE). The cadavers belonged to the Department of Anatomical Dissection and Donation of the Medical University of Lodz, Poland.

### 2.1. Statistical Analysis

The Chi^2^ test was used to compare differences in the numbers of biceps brachii bellies according to body side and sex. The Shapiro-Wilk was used to evaluate the normality of the distribution of continuous data. As data was not normally distributed, the Mann–Whitney test was used to compare continuous data between sexes and the Wilcoxon test between body sides. The Mann–Whitney test and the Kruskal-Wallis test by ranks with dedicated *post hoc* test were used to compare measurements between biceps brachii muscle with different numbers of bellies.

Statistical analyses were performed using Statistica 13.1 (StatSoft, Cracow). A *p* value lower than 0.05 was considered significant.

## 3. Results

Standard anatomy of BB muscle bellies was observed in 59% of dissected limbs (26 females and 33 males; 29 right and 30 left).

**Figure 1:**
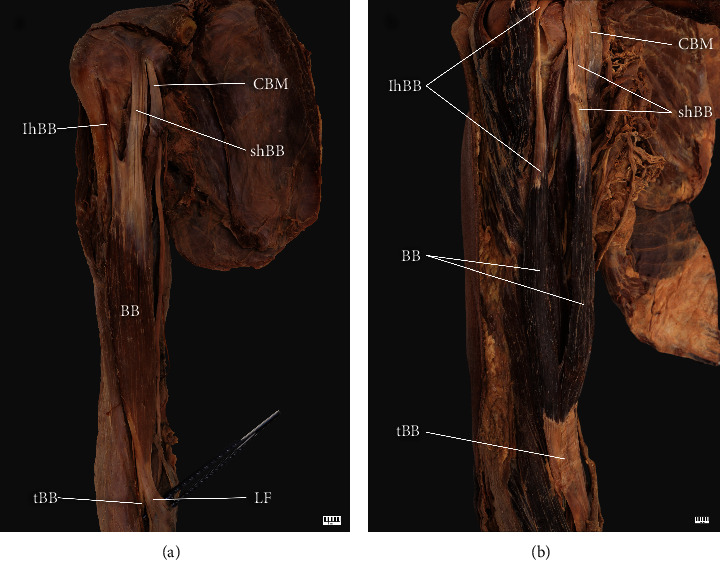
Type I of the biceps brachii. (a) Type Ia of biceps brachii. Right arm: *CBM* coracobrachialis muscle; *shBB* short head of the biceps brachii; *lhBB* long head of the biceps brachii; *BB* biceps brachii; *tBB* tendon of the biceps brachii; *LF* lacertus fibrosus. (b) Type Ib of biceps brachii. Right arm: *CBM* coracobrachialis muscle; *shBB* short head of the biceps brachii; *lhBB* long head of the biceps brachii; *BB* biceps brachii; *tBB* tendon of the biceps brachii.

Type I (64%) has two heads: long and short. This type was divided into two subtypes (A-B) according to the number of bellies:

- Single muscle belly. The fibers derived from the long head of biceps fuse with a short head at the lower end of the intertubercular groove. This type was found in 5 cases (5 men; 3 right and 2 left) (Figure 1(a))
- Two muscle bellies. Standard anatomy of the BB. Both heads of the BB join in the middle upper arm to form a muscle mass. This type was present in 59 limbs (26 females and 33 males; 29 right and 30 left) (Figure 1(b))

**Figure 2:**
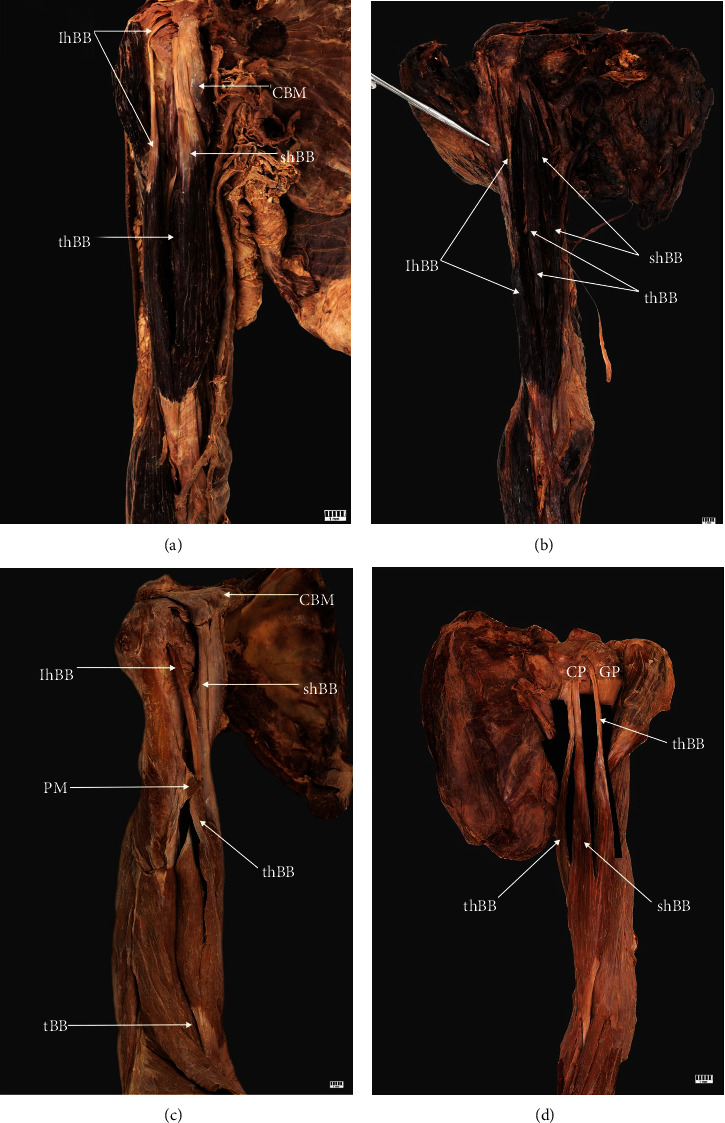
Type II of the biceps brachii. (a) Type IIa of biceps brachii. Right arm: *CBM* coracobrachialis muscle; *shBB* short head of the biceps brachii; *lhBB* long head of the biceps; *thBB* third head of the biceps brachii. (b) Type IIb of biceps brachii. Right arm: *shBB* short head of the biceps brachii; *lhBB* long head of the biceps brachii; *thBB* third head of the biceps brachii. (c) Type IIc of the biceps brachii. Right arm: *shBB* short head of the biceps brachii; *lhBB* long head of the biceps brachii; *thBB* third head of the biceps brachii; *PM* tendon of the pectoralis major muscle d Type IId of the biceps brachii. Left arm: *CP* coracoid process; *GT* greater tubercle; *lhBB* long head of the biceps brachii; *shBB* short head of the biceps brachii; *thBB* third head of the biceps brachii.

Type II (26%) has three BB heads. This type was observed in 26 upper limbs (16 women and nine men, 12 right and 14 left). The short head originates from the coracoid process, while the long head originates from the supraglenoid tubercle. This type was divided into four subtypes based on the site of attachment of the third head (A-D):

- The third head attached to the middle part of the shaft of the humerus. This subtype was observed in 14 upper limbs (Figure 2(a))
- The third head was an accessory head of the short head of the BB. It originated on the coracoid process, together with the short head of the BB. This subtype was found in four cases (Figure 2(b))
- The third head originated from the pectoralis major muscle. This subtype was recognized in two arms (Figure 2(c))
- The third head originated from the capsule of the humeral joint. This subtype was found in six cases (Figure 2(d))

**Figure 3:**
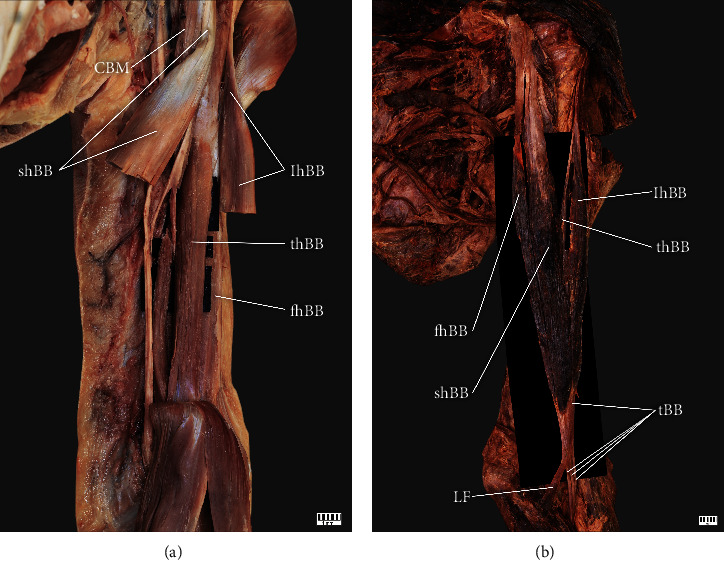
Type III of the biceps brachii. (a) Type IIIa of biceps brachii. Left arm: *CBM* coracobrachialis muscle; *shBB* short head of the biceps brachii; *lhBB* long head of the biceps brachii; *thBB* third head of the biceps brachii; *fhBB* fourth head of the biceps brachii. (b) Type IIIb of the biceps brachii. Left arm: *CBM* coracobrachialis muscle; *shBB* short head of the biceps brachii; *lhBB* long head of the biceps brachii; *thBB* third head of the biceps brachii; *fhBB* fourth head of the biceps brachii; *tBB* tendon of the biceps brachii; *LF* lacertus fibrosus.

Type III (6%) has four heads of the BB. This type was observed in 6 upper limbs (4women and 2 men, 3 right and 3 left). The short head originated from the coracoid process, while the long head originated from the supraglenoid tubercle. This type was divided into two subtypes (A-B) based on the attachments of the third and fourth heads:

- Type IIIa – four cases: Both the third and fourth head of the BB originates from the humerus bone (Figure 3(a))
- Type IIIb – two cases: The third head originates from the short head of the BB, while the fourth head originates from the long head of the BB (Figure 3(b))

**Figure 4:**
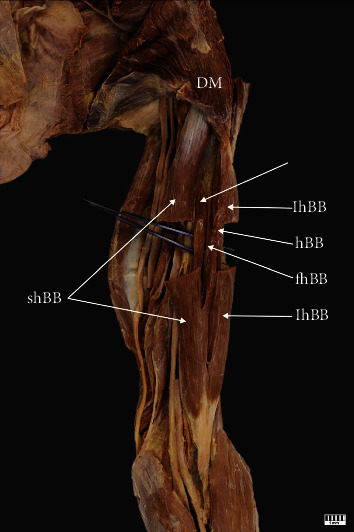
Type IV of the biceps brachii. Left arm: *DM* deltoideus muscle; *lhBB* long head of the biceps brachii; *shBB* short head of the biceps brachii; *thBB* third head of the biceps brachii *fhBB* fourth head of the biceps brachii; *5hBB* fifth head of the biceps brachii.

Type IV (4%) has five heads of the BB. This type was found in 4 upper limbs (1 woman, 1 man, 2 right and 2 left). The short head originates from the coracoid process, while the long head originates from the supraglenoid tubercle. Both the third and fourth head originate from the shaft of the humerus, while the fifth head originates from the pectoralis major muscle (Figure 4).

According to Chi^2^ test, the difference in gender distribution borderline is significant (*P* = 0.0519), while differences in body sides were not significant (*P* = 0.9933).

The morphological parameters are compared between body sides and sexes in Table 1 and according to the belly number in Table 2.

According to the post hoc analysis:

- Proximal LHBT length was greater in muscles with fife bellies than in all other types
- Width of LHBT-belly junction was larger in muscles with two and three bellies than in remaining types
- Thickness of LHBT-belly junction was larger in muscles with two, three, and five bellies than in type with just one belly
- Proximal SHBT length was lower in muscles with three bellies than in remaining types
- Length of LHB was lower in muscles with two bellies than with three bellies
- Length of 3^rd^ head was greater in muscles with three bellies than with four and five
- Width of SHBT-belly junction was larger in muscles with two and three bellies than in types with one and five bellies. Additionally muscles with four bellies had significantly wider SHBT-belly junction than in muscles with one head
- Thickness of SHBT-belly junction was larger in muscles with two and three bellies than in types with one and four bellies

## 4. Discussion

In addition to the large body of work performed on the accessory heads of the BB, the present work provides a new systematic classification of accessory heads of the BB based on anatomical dissection. It should be emphasized that this is the first classification of this type and may be of value for orthopedists and surgeons operating in this area. It can also be useful for physiotherapists when planning rehabilitation procedures and radiologists for imaging analysis.

To understand the occurrence of the accessory head of the BB, it is necessary to review its embryological development. The BB, coracobrachialis, and brachialis are intimately fused together in very early stages and probably arise from a common premuscle mass. At this early stage, the two heads of the BB are located close together and only become separated by the later growth of the scapula. The three muscles can be recognized in embryos measuring 14 to 16 mm in length, and the long tendon of the caput longum can be recognized in embryos 14 mm long. The distal end of the common muscle mass differentiates later than the proximal end [26]. The third, fourth, and fifth heads of BB are believed to arise from the brachialis muscle, and in such instances, the distal attachment has been translocated from the ulna and radius [27].

The BB appears to be one of the most morphologically variable muscles in the upper limb, matching the variability of the coracobrachialis muscle and subscapularis muscle [18, 23–25, 28]. Although it is rare for the short or long head of the BB to be absent or for their origin and insertion to vary [12, 15–17, 27], supernumerary heads are relatively frequent, with perhaps three, four, or even five heads present [2, 3, 7, 10, 14, 29–31]. A wide range of classification systems and case reports have been created on this basis; however, there is a need to gather them into a uniform, systematic classification.

Regarding the supernumerary heads of BB, the most common variation involves the presence of a third head, which ranges from 4.2% to 19.8% [2, 3, 10, 16, 27, 29–32].

Morphological variations in the form of supernumerary heads were first described by Testut [27], MacAlister [33], Le Double [16], Anson [34], and Bergmann [15]; however, they were first included in a classification by Kosugi et al. [10], who classified both types with a third head and types with a fourth. The types with a third head were subdivided into Type a, running from the shaft of the humerus between the coracobrachialis and brachialis muscle; Type b, running from the medial brachial intermuscular septum, and Type c, running from other regions, e.g. the tendon of the pectoralis major, deltoid muscle or greater tubercle, or even the articular capsule.

Alternatively, Rodriqiguez-Niedenfuhr et al. describe three possibilities of proximal attachment of the third head of the BB [31]. The most common type (31 cases; 9%) was the inferomedial humeral head, characterized by a flat muscular belly with a proximal attachment to the anteromedial surface of the humerus; it was continuous with the insertion of the coracobrachialis muscle and was closely related to the medial intermuscular septum and brachialis muscle. In the Type V (5 cases; 1.5%), was superior humeral head was a slender and flat muscular slip that had a proximal attachment to the surface of the humerus between the lesser tubercle and the origin of the coracobrachialis and brachialis muscle. The rarest type (1 case; 0.3%) was the presence of an inferolateral humeral head, which arose from the lateral intermuscular septum between the insertion of the deltoid muscle and the origin of the brachioradialis muscle [31]. The differences between these studies on the additional third head are presented in Table 3.

The presence of a fourth head is much rarer than of a third head [10, 16, 31, 35]. Kosugi et al. [10] report that the fourth head of the BB may originate from the pectoralis major tendon, greater tubercle, or articular capsule. The differences between existing research on the additional fourth head are presented in Table 4.

In turn, the presence of fifth head of the BB has been very rarely described [31].

As the classification by Kosugi et al. [10] seems to be very inaccurate, and all other works are solely based on the variability of only the third head, we propose a four-folded classification. In our classification, Type I, the most frequent type (64%), is characterized by two heads, one long and one short. This type is divided into two subtypes (A-B) according to the number of bellies. The second most common type (26%) was Type II, which was characterized by three BB heads; as the third head demonstrated a range of attachment sites, this type was divided into several subtypes (A-D). Unlike Kosugi et al. [10] and Rodriguez et al. [31], our proposed classification details the exact locations of the proximal attachment; Kosugi et. [10] which assumes a type c - different place, is definitely inappropriate.

Our proposed types, together with the subtypes, determine the exact proximal attachment sites of the third head of the BB. The suggested division may be of great assistance for surgeons operating in this area, as well as radiologists performing imaging assessment. The third most common type was Type III (6%) which was characterized by four BB heads. This type was divided into two subtypes (A-B) due to the fact that the third and fourth heads had different sites of attachment. Interestingly, this type was rarely described by other authors. In our study, it constitutes 5% of all samples and has been divided into subtypes. Type IV (4%) was characterized by five heads. Both the third and fourth head originate from the shaft of the humerus, while the fifth head originates from the pectoralis major muscle.

The presence of supernumerary heads is an important consideration when assessing BB function and potential dysfunction such as plural injury. Additionally, these variations are important for surgeons, when there is an unusual bone displacement subsequent to humeral fracture due to the alteration of the biomechanical forces applied to the humerus. Accessory heads would be expandable and possibly have more value in flap surgery by plastic surgeons; however, supernumerary heads may compress neurovascular structures because of their close relationship with the brachial artery and musculocutaneous, median nerve, and such impingement that may lead to unexplained upper limb and shoulder pain syndromes and nerve compression syndromes.

The present study has some limitations. The classification is quite heterogeneous and depends on several morphological details, such as the number of supernumerary heads of the BB. In addition, as it is only an anatomical study, a spectrum of variation could be presented. Further studies should examine the potential value of ultrasound or MRI for this purpose. Nonetheless, this work helps raise awareness of what to look for, and where to find it. It also offers a uniform classification and terminology which can be used as a foundation for communication with surgeons.

The indication of BB variants may direct the light on importance in terms of recognizing the cause of damage/pressure on, for example, the brachial artery, branches of the musculocutaneous nerve, and its branches. In the case of damage to the BB muscle, it also indicates the need to carefully check the variance of this muscle before planning the treatment procedure.

Due to the presence of a greater number of bellies, the number of trigger points also changes, which brings the need for more precise mapping for treatment. Also, the work on the BB fascia, depending on the variant, should be adapted and not assumed work as in the case of standard two bellies.

## 5. Conclusion

The biceps brachii is characterized by very high morphological variability. The new classification proposes four types of the supernumerary heads of biceps brachii (I-IV). As types have been divided into subtypes, the classification has both clinical and anatomical significance.

## Figures and Tables

**Table 1:** Morphometric parameters according to body sides and genders. Data presented as mean (standard deviation).

Parameter	Body side		*P* value^1^	Gender		*P* value^2^
	Right	Left		Females	Males	
Proximal LHBT length	137.9 (27.2)	138.9 (27.2)	0.8966	133.5 (27.5)	143.9 (25.7)	0.2418
Width of LHBT-belly junction	12.1 (4.2)	12.0 (4.2)	0.8776	12.1 (4.5)	11.9 (3.9)	0.9885
Thickness of LHBT-belly junction	2.3 (0.8)	2.2 (0.9)	0.6406	2.4 (0.8)	2.2 (0.9)	0.4210
Acc. LHBT length	60.7 (16.7)	58.6 (15.7)	0.6726	55.1 (17.1)	61.2 (15.7)	0.5913
Width of acc. LHBT-belly junction	3.3 (0.8)	3.4 (0.9)	1.0000	3.7 (0.4)	3.3 (0.9)	0.4779
Thickness of acc. LHBT-belly junction	1.5 (0.4)	1.6 (0.5)	0.8501	2.0 (0.3)	1.4 (0.4)	0.0421
Proximal SHBT length	81.7 (22.1)	80.2 (18.9)	0.9272	81.9 (20.3)	79.8 (20.8)	0.4437
Width of SHBT-belly junction	12.1 (4.5)	11.7 (4.5)	0.6790	12.3 (4.9)	11.5 (4.0)	0.9501
Thickness of SHBT-belly junction	2.3 (0.9)	2.1 (0.8)	0.2501	2.3 (0.7)	2.0 (1.0)	0.0770
Length of LHB	152.8 (25.9)	153.9 (28.0)	0.9673	140.7 (27.2)	166.4 (19.3)	0.0001
Length of SHB	165.5 (34.4)	164.2 (37.8)	0.9081	153.3 (30.4)	176.7 (37.5)	0.0005
Length of 3^rd^ head	94.7 (60.1)	105.3 (62.4)	0.6693	118.3 (44.6)	71.1 (72.2)	0.1232
Length of 4^th^ head	110.9 (28.6)	123.1 (39.9)	0.9273	125.4 (7.5)	108.2 (51.4)	0.2353
Length of 5^th^ head	111.1 (1.6)	110.2 (0.0)	0.6831	110.1 (0.2)	111.2 (1.4)	0.4142
Width of distal myotendinous junction	11.8 (3.2)	12.0 (3.5)	0.7020	10.8 (3.0)	12.9 (3.4)	0.0045
Thickness of distal myotendinous junction	3.2 (0.9)	3.3 (1.2)	0.7661	3.1 (1.2)	3.3 (0.9)	0.0751

^1^The Wilcoxon test; ^2^The Mann–Whitney test.

**Table 2:** Morphometric parameters according to number of biceps brachii muscle bellies. Data presented as mean (standard deviation).

Parameter	Number of biceps brachii muscle bellies					*P* value^1^
	1 (*n* = 5)	2 (*n* = 59)	3 (*n* = 26)	4 (*n* = 6)	5 (*n* = 4)	
Proximal LHBT length	133.7 (17.8)	138.5 (30.7)	138.4 (15.8)	111.8 (14.3)	183.6 (7.2)	0.0014
Width of LHBT-belly junction	6.2 (1.7)	13.3 (3.5)	13.4 (3.4)	5.1 (0.6)	8.4 (0.4)	0.0001
Thickness of LHBT-belly junction	1.3 (0.1)	2.3 (0.9)	2.5 (0.9)	1.8 (0.5)	2.3 (0.1)	0.0369
Acc. LHBT length		68.3 (13.9)	45.8 (5.3)			0.0021
Width of acc. LHBT-belly junction		3.0 (0.8)	3.8 (0.5)			0.0277
Thickness of acc. LHBT-belly junction		1.4 (0.5)	1.8 (0.4)			0.0586
Proximal SHBT length	79.8 (7.2)	86.0 (18.5)	69.1 (24.1)	93.3 (2.5)	87.7 (1.9)	0.0086
Width of SHBT-belly junction	4.2 (0.2)	13.7 (4.1)	12.2 (3.2)	9.7 (3.4)	5.4 (0.2)	0.0001
Thickness of SHBT-belly junction	1.1 (0.1)	2.3 (0.8)	2.4 (0.8)	1.3 (0.1)	2.0 (0.1)	0.0011
Length of LHB		158.9 (26.9)	140.9 (27.6)	154.9 (11.8)	150.4 (1.6)	0.0211
Length of SHB		164.6 (38.8)	168.8 (34.3)	146.3 (18.9)	170.6 (6.6)	0.1773
Length of 3^rd^ head			82.9 (63.4)	137.3 (6.8)	154.7 (1.8)	0.0063
Length of 4^th^ head			185.5 (0.0)	107.1 (35.7)	116.2 (1.0)	0.2116
Length of 5^th^ head					110.7 (1.0)	—
Width of distal myotendinous junction	12.9 (2.6)	11.8 (3.4)	11.6 (3.8)	11.3 (1.0)	14.7 (1.0)	0.3414
Thickness of distal myotendinous junction	3.5 (0.4)	3.2 (1.2)	3.1 (0.9)	3.8 (0.8)	3.4 (0.4)	0.2927

^1^The result of ANOVA omnibus test; LHBT: long head of the biceps brachii; SHBT: short head of the biceps brachii tendon.

**Table 3:** Difference between study of the presence of the third head of the biceps brachii.

Study	Number of cases	Percentage
Kosugi et al. [10]	75	13.7
Rodriguez-Niedenfuhr et al. [31]	27	7.7
Nayak et al. [30]	2	4.2
Bellasteros etl al. [3]	21	19.8
Al-Kushi et al. [2]	6	15
Present study – Szewczyk et al.	25	31.25

**Table 4:** Difference between presence of the fourth head of the biceps brachii.

Study	Number of cases	Percentage
Kosugi et al. [10]	7	1.3
Rodqigues-Niedenfuhr et al. [31]	5	1.4
Present study – Szewczyk et al.	6	7.5

## Data Availability

Please contact the authors for data requests (Łukasz Olewnik PhD; email address: lukasz.olewnik@umed.lodz.pl).
